# Experimental changes in food and ectoparasites affect dispersal timing in juvenile burrowing owls

**DOI:** 10.1371/journal.pone.0306660

**Published:** 2024-07-26

**Authors:** Victoria Garcia, Courtney J. Conway, Christopher P. Nadeau

**Affiliations:** 1 Arizona Cooperative Fish and Wildlife Research Unit, School of Natural Resources and the Environment, University of Arizona, Tucson, Arizona, United States of America; 2 United States Geological Survey, Idaho Cooperative Fish & Wildlife Research Unit, University of Idaho, Moscow, Idaho, United States of America; Cairo University Faculty of Veterinary Medicine, EGYPT

## Abstract

Natal dispersal is a key demographic trait that affects population dynamics, and intraspecific variation in dispersal affects gene flow among populations and source-sink dynamics. However, relatively little is known about the selective pressures and trade-offs that animals face when departing their natal area due to the logistical difficulties associated with monitoring animals during this critical life stage. We used a randomized block design to examine the selective pressure that influence dispersal timing in juvenile burrowing owls (*Athene cunicularia*) by experimentally altering both food and ectoparasites at 135 nests. We also examined the effects of local food abundance, ectoparasite loads, and parental departure on natal dispersal timing. Juvenile burrowing owls varied widely in natal dispersal timing, and phenotypic plasticity in dispersal timing was evident in juvenile owls’ response to our experimental treatments, local conditions, and their parents’ departure from the natal area. Moreover, juveniles responded differently than their parents to experimental manipulation of food and ectoparasite loads. Juveniles typically dispersed shortly after their parents departed the natal area, but delayed dispersing more than 2 weeks after parental departure if they did not receive experimental food supplements during a low-food year. In contrast, the experimental food supplements did not affect the migratory departure decisions of adult owls in either year. Juveniles at nests treated for ectoparasites initiated dispersal at a younger age (and *prior* to adults in the high-food year) compared to juveniles at control nests. In contrast, parents at nests treated for ectoparasites departed later than parents at control nests. Our results suggest that unfavorable conditions (low food or high ectoparasite loads) caused juveniles to delay dispersal, but prompted adults to depart sooner. Our results highlight the extent of intraspecific variation in natal dispersal timing, and demonstrate that ecological conditions affect dispersal decisions of parents and offspring differently, which can create important trade-offs that likely affect life history strategies and responses to climatic changes.

## Introduction

Dispersal is a key demographic trait that helps animals persist in the face of spatial stochasticity in habitat quality, facilitates colonization of new areas, and maintains genetic diversity and gene flow. Natal dispersal is vital to many population viability models [1] and is an important life history trait that affects population dynamics [2, 3]. The age at which juveniles leave their natal area is the first in a series of decisions that juveniles make after they gain independence. The age at which juveniles initiate natal dispersal is an important event in an animal’s life cycle, and yet natal dispersal timing varies substantially among species [4], across latitudinal gradients [5], and even within broods [6, 7]. Variation in natal dispersal timing is associated with differences in breeding strategies [8, 9] and allocation of parental effort [10, 11] and, hence, is part of a species’ life history strategy. Intraspecific variation in natal dispersal timing has been well-studied in communal breeders [12, 13], but has received less attention in species that do not breed communally. Many species exhibit delayed dispersal but do not breed communally, suggesting that delayed dispersal might be beneficial under some ecological conditions even without the potential benefits of becoming a helper [12, 14, 15]. Hence, the factors that influence variation in natal dispersal timing (i.e., why delay dispersal?) may not be the same as those that influence variation in helping behavior and breeding strategies [14, 16–18].

Relatively few studies have documented intraspecific variation in the age at which juveniles initiate natal dispersal, especially in species that do not breed communally [12, 19–24] and even fewer have experimentally tested multiple hypotheses to explain the cause of intraspecific variation in timing of natal dispersal. Onset of natal dispersal can be affected by sibling competition, parent-offspring conflict, population density, body condition, age of parents, and resource availability [12, 20, 25–29]. Moreover, intraspecific variation in natal dispersal timing may reflect life history trade-offs of parents (i.e., the timing of parental departure might affect the timing of juvenile dispersal) and also affects juvenile survival in many species [30, 31] and, hence, lifetime fitness of the parents.

We documented the extent of intraspecific variation in natal dispersal timing of western burrowing owls (*Athene cunicularia hypugaea*) and tested whether two processes explain variation in dispersal timing: food abundance and ectoparasite load. Intraspecific variation in natal dispersal timing has not been well-documented in burrowing owls, but available evidence suggests that individuals vary substantially: some juveniles leave their natal area soon after they can fly (~40 days old) and others remain for over twice that long [7, 32]. Intraspecific variation in natal dispersal timing is relatively easy to document in burrowing owls because fledglings continue to perch at or near their natal nest burrow (to roost and escape predators) until they initiate natal dispersal movements [33]. Burrowing owls are ideal for testing whether local food abundance and ectoparasites affect natal dispersal timing because intraspecific variation in dispersal can be determined within a single year (only 0.3% of juveniles in our study area in eastern Washington overwintered; [34]), and natal dispersal is not confounded by natal philopatry (almost all juveniles make their first breeding attempt at a burrow other than their natal burrow; [33]). Moreover, burrowing owl populations have declined in many portions of their range [35–37], dispersal causes genetic differentiation among populations [38], and mortality during the post-fledgling/fall dispersal period is thought to have large influences on local population trends and population dynamics [32, 39].

Burrowing owls eat small rodents and invertebrates, and the abundance of these prey items can vary seasonally (Wellicome 2000). Also, many burrowing owls have ectoparasites (fleas and lice) [40–42], but the prevalence of ectoparasites varies widely among individuals both among and within years [43]. This variation in food abundance and ectoparasite loads may cause intraspecific variation in natal dispersal timing. Abundant food could either cause birds to: 1 fledge early (develop faster) [44], or 2) stay with parents longer to take advantage of feeding [45–47]. High ectoparasite loads could cause juveniles to either leave early (to escape the parasite burden) or stay (if high parasite loads slow development). To examine how food and ectoparasites influence timing of natal dispersal, we manipulated food and ectoparasites at burrowing owl nests to test whether natal dispersal timing was phenotypically plastic (i.e., whether natal dispersal timing was responsive to changes in environmental conditions), and, if so, which of two potential causal mechanisms explain more of the intraspecific variation: food or ectoparasites. We also measured ambient levels of prey availability and ectoparasite loads at a subset of nests because the effectiveness of any experimental manipulation at influencing behavioral traits is undoubtedly affected by ambient levels of those resources (i.e., experimentally adding food when food in the landscape is already abundant is less likely to alter an animal’s behavior even if food influences that behavior). We also examined the effects of our experimental treatments on parental departure (and the effects of parental departure on natal dispersal timing) so that we could separate the direct and indirect effects of our food and ectoparasite experimental treatments (i.e., whether food and ectoparasites influence natal dispersal timing directly or merely through their effects on parental departure).

## Materials and methods

### Study species

Western burrowing owls are small (~150 g) owls that nest in burrows dug by burrowing mammals such as prairie dogs (*Cynomys* spp.) and badgers (*Taxidea taxus*) [37]. Burrowing owl eggs (4–12 eggs/clutch) hatch asynchronously, with 2 or more eggs typically hatching on the same day and the remaining eggs hatching during the subsequent 4–5 days [48–50]. Burrowing owls are not territorial, but adults will vigorously defend their own burrows from other adults [33,51]. Recruitment of local juveniles into the breeding population is typically low; -4-8% of juveniles were recruited into the local breeding population at 2 study sites in eastern Washington based on 4 years of intensive field work at those sites [34].

### Study area

Our study area covered approximately 3600 km^2^ of irrigated croplands and sagebrush steppe in central Washington (Grant and Adams counties). In this area, elevation varies from 316–398 m above sea level and annual precipitation is usually <25 cm, which falls primarily as rain from October to May [52]. Most adults (and virtually all juveniles) migrate away from the area for the winter [34]. We used 3 approaches to locate burrowing owl nests: roadside surveys [53], incidental sightings, and talking to landowners. We visited each nest once or twice per week from March through mid-September in both 2002 and 2003.

All methods and activities were approved by the Washington Department of Fish and Wildlife (WDFW #04–1020), the U.S. Department of the Interior (Federal Bird Banding Permit #22524), and the University of Arizona’s Institutional Animal Care and Use Committee (approved protocols #01–089 and #03–052). Landownership was a combination of County, private, and federal lands and we had permission to conduct the field work from all landowners where we worked.

### Effect of food and ectoparasite treatments on natal dispersal timing

We assigned two treatments to nests in a full factorial arrangement with a completely randomized design. Each nest was assigned to one of four experimental treatment groups: 1) nests which received food and ectoparasite treatments, 2) nests which received only food treatments, 3) nests which received only ectoparasite treatments, or 4) nests which received neither treatment.

At food-supplemented nests (those in groups 1 and 2 above), we provided 95–105 g of dead laboratory mice per owl every 7 days (≥13.6 g of food per owl per day), which constituted ≥50% of the family’s (adults and juveniles) weekly energetic needs [54]. We placed mice inside the entrance to all burrows used by adults and juveniles. We often saw both adult and juvenile burrowing owls eat or cache the supplemented mice.

We applied insecticide (diatomaceous earth powder without pyrethrin) to nests (those in groups 1 and 3 above) every 7 days to reduce ectoparasite loads. Diatomaceous earth can be used to control internal and external parasites in livestock and pets. For example, diatomaceous earth reduced ectoparasites in nests of tree swallows (*Tachycineta bicolor*) [55] and is used to control ectoparasites in chicken coops and chinchilla (*Chinchilla lanigera*) pens by making it available for dust baths [56, 57]. We placed 100 ml of diatomaceous earth powder inside the entrance of each ectoparasite-treatment burrow during weekly nest visits so that owls could take dust baths [58] with the powder. We also sprayed the tunnel of the nest burrow 5 times each visit (to saturate the dirt for a brief time) with a solution of water and diatomaceous earth. We treated a subset of nests with diatomaceous earth explicitly to determine whether reduction of ectoparasites would affect timing of natal dispersal, but we did not notice any unintended side effects of these treatments (e.g., the treatments did not appear to alter immediate behavior of burrowing owls and did not decrease the number of offspring at each nest prior to dispersal).

We first provided supplemental food and diatomaceous earth to experimental nests after juveniles initially emerged from their nest burrow (typically 14 days old; [59–62]), and we continued to treat nests until no juveniles were present within the natal area (i.e., the area within 300 m of the natal burrow; [7]). Therefore, all the juveniles at treated nests received treatments from the time they were ~14–21 days old until they dispersed from their natal area. We applied treatments to nests every 7 days during weekly nest visits to all nests so that experimental nests were not visited more frequently than control nests. We could not directly evaluate whether the treatments affected juvenile body condition due to variation in the ages that we captured juveniles. That is, we captured some juveniles soon after (or even before) their nest received their first treatment, whereas others had been treated for weeks prior to capture.

We determined natal dispersal timing by placing a 4.6-g VHF radio transmitter (Model PD-2C, Holohil Systems Ltd., Ontario, Canada) on one juvenile burrowing owl in each brood. We attached radio transmitters to 146 juveniles from 135 broods (67 in 2002 and 68 in 2003). We often caught several juveniles from a nest at the same time, and the transmitter was placed on the first one that we pulled out of the trap. We attached aluminum leg bands to all captured juveniles. If a radio-collared juvenile died, we attempted to trap and radio-collar a banded sibling from the same brood (i.e., we used only 1 juvenile per brood and treated juveniles as independent sampling units in our analysis). Radio-collars do not influence behavior or survival of juvenile burrowing owls [63].

We used a handheld 3-element Yagi antenna to locate all radio-collared juveniles every 2–3 days. When we could not locate a radio-collared juvenile for 4 days, we used a vehicle-mounted whip antenna to scan for the missing signal while driving throughout a 3.2-km^2^ area surrounding the location where that juvenile was last detected. If vehicular scans of the surrounding location were unsuccessful, we searched for the missing signals from a fixed-wing airplane flown over and beyond the boundaries of the study area. We scanned all the missing frequencies during each flight, allowing us to locate 39 of 66 owls that could not be located from the ground. We excluded from our analyses the juveniles that died prior to dispersal and birds whose signal we could not locate after vehicle and aerial searches because we could not be certain whether they had died near the natal area (and a predator destroyed the transmitter) or they dispersed well beyond the study area. We assumed a juvenile had initiated natal dispersal when the juvenile roosted >300 m (King and Belthoff 2001) away from the natal burrow for 2 or more consecutive visits (63 of the 146 radio-marked juveniles survived through dispersal and we maintained contact with them after initiating dispersal). Only 3 of these 63 juveniles returned to roost near their natal burrow after having roosted >300 m from their natal burrow for 2 consecutive visits (i.e., initiated dispersal). Because we could not visit each nest site daily, we recorded dispersal date for each dispersed juvenile as the mid-point between the last date the juvenile was documented present in the natal area and the first date the juvenile was no longer present in the natal area (i.e., the mid-point of the final 2–3 day interval).

We estimated the initiation of hatching for each brood and included hatch date in our analysis because we thought that juveniles from breeding attempts initiated later in the breeding season might disperse at a younger age regardless of our experimental treatments. We calculated the age of the juvenile on the date it left the natal area (natal dispersal timing) by subtracting its estimated hatch date from its estimated dispersal date. Because burrowing owl hatch dates in natural burrows are difficult to observe directly, we used a protocol to estimate the date the first group of eggs in each brood hatched [62]. The protocol uses the following resources to estimate hatch date: a photographic guide for aging nestlings (modified from [64]), partial and final clutch sizes recorded with an infrared video probe (Sandpiper Technologies Inc., Manteca, CA) during weekly nest visits, average laying interval [48], and average incubation period [33]. Estimates of hatch date based on the protocol proved accurate when we compared them to known hatch dates at a subset of nests where infrared video documented actual hatching dates.

We recorded parental departure date as the mid-point between the date of the last nest visit when ≥1 adult was seen in the natal area and the date of the first nest visit when no adult was subsequently seen in the natal area. We then calculated the age of the juvenile on the estimated date of parental departure by subtracting the juvenile’s hatch date from the date of parental departure. We were unable to estimate a parental departure date for 1 juvenile which we subsequently excluded from the parental departure analyses. In our study, we defined parental departure as the age of the juvenile when its last parent had left the natal area. After adults left the natal area, we were unable to determine whether they had moved to another burrow within our study area or left the study area completely (i.e., initiated fall migration).

Eighty-eight (70%) of the 126 possible adults at the 63 focal nests were banded with a unique color-band. None of the pairs of banded adults nested together during both years of the study and only 8 of these 88 adults were known to be present in both years of the study. Of these 8 adults, only 2 were at the same burrow both years. Thus, we treated each adult (and its brood) as independent in our analysis because: 1) few adults (and no pairs) were present during both study years, 2) the treatments were assigned randomly each year regardless of adult identity, and 3) 30% of the 126 adults were not banded.

### Statistical analysis of food and ectoparasite experimental treatments

Because our study area was so large (~3600 km^2^) and included noticeable clusters of breeding pairs, we divided the study area into smaller geographic units (regions) that had similar elevation, topography, land uses, and breeding phenology. We used generalized linear mixed models (GLMM) to examine the effects of the two experimental treatments on the following response variables: natal dispersal timing, parental departure (i.e., juvenile age on the date parents departed the natal area), and the difference (in days) between natal dispersal timing and parental departure. We used a two-stage approach to identify the factors affecting each response variable. During the first stage, we developed a set of *a priori* candidate models that only included potential covariates (variables that we thought might affect dispersal) including: region (random effect), number of nestlings in each brood 30 days after hatching (brood size), hatch date, parental departure (only for analyses of natal dispersal timing), and 2-way interactions that we thought important. We used Restricted Maximum Likelihood (REML) estimators in R [65] to estimate variance components. We used generalized linear models and maximum likelihood estimators [66] to assess models based on AICc (Akaike’s Information Criterion corrected for small sample size). For each of the 3 response variables, we chose the model with the lowest AICc value from among the candidate model set and used the variables from that top model in the second stage. In the second stage for each of the 3 response variables, we developed another set of candidate models that included the covariate(s) from the top model of the first stage, the main effects (our 2 experimental treatments), year (2002 or 2003), and 2-way interactions. When no single model carried the preponderance of evidence, we used Package AICcmodavg [67] in R for model averaging. Group means presented in graphs are the model-averaged predicted values ±1 unconditional standard error or the predicted values ±1 standard error from the top model, depending on whether we model-averaged across the model set or not.

### Ambient food abundance and ectoparasite loads

Abundance of burrowing owl prey items can vary seasonally and across years [68, 69]. Therefore, we estimated local food abundance each year to determine whether ambient food abundance (prey availability) in the landscape differed between the 2 study years, whether it increased or decreased throughout the breeding season each year, and whether ambient food abundance was correlated with natal dispersal timing. Because small mammals comprise ~90% of the biomass of burrowing owl diets [70], we trapped small mammals at nest burrows that did not receive supplemental food to provide an estimate of relative food abundance. We trapped rodents only at burrows that did not receive food supplements because we thought the constant addition of food to food-supplemented burrows throughout the fledging and post-fledging period could result in decreased hunting around the burrow (and possibly even attract some animals) and therefore result in biased estimates of local food abundance. In 2002, we trapped small mammals at all the owl nest burrows that did not receive supplemental food. In 2003, we trapped small mammals at half the nest burrows that did not receive supplemental food so that we could examine whether our small mammal trapping effort influenced natal dispersal timing of juvenile burrowing owls (it did not; *P* = 0.367). We randomly selected the subset of burrows at which we trapped small mammals in 2003 from among all nests that did not receive food supplements. We only trapped small mammals at each nest for a total of 4 nights (2 trapping sessions with 2 trapping nights/session and >4 weeks between the 2 trapping sessions) and we released all rodent captures on-site, so we do not believe that our trapping efforts significantly reduced prey availability or affected natal dispersal timing of owls.

We used live-traps baited with peanut butter and rolled oats to trap small mammals, and we set the trap arrays on two occasions at each nest: 1) just prior to the age when juveniles at that nest would begin dispersing (~40 days old), and 2) late in the breeding season, when many juveniles were initiating dispersal (~85 days old). We set 64 traps with 15-m spacing between traps in an 8×8 grid centered on each of 49 nest burrows. We cut the tip of one ear or a patch of fur above the hind legs on all captured rodents so that we could exclude recaptures and we summed the number of rodents captured in the 64 traps during each of the two trapping sessions as our index of food abundance.

We measured ectoparasite loads of juvenile owls at nests to determine relative levels of ectoparasite (mostly fleas) infestation, and to test whether ectoparasite load was correlated with natal dispersal timing. We assigned each juvenile an index of ectoparasite abundance (0–5) at time of capture. We assigned owls a 0 if no ectoparasites were visible on the skin or feathers, a 1 if ~1–2 ectoparasites were visible, a 2 if ~3–6 ectoparasites were visible, a 3 if ~7–10 ectoparasites were visible, a 4 if ~11–15 ectoparasites were visible, and a 5 if >15 ectoparasites were visible. We used the average ectoparasite loads among all juveniles (all siblings) to obtain one value for each nest burrow and used those nest averages to examine whether ectoparasite loads differed between our 2 study years.

### Statistical analysis of ambient food and ectoparasite data

We used GLMMs in R to determine whether ambient food abundance differed between the two years. The explanatory variables were: nest as a random factor to account for the two trapping sessions at each nest, trapping session (1^st^ or 2^nd^) as a fixed factor, year, and an interaction between year and trapping session. We compared models that included year and year*trapping session (in addition to nest) with models that only contained trapping session and nest, or nest alone. We used AICc to compare candidate models. Comparing these models allowed us to evaluate whether local food abundance differed between years or between trapping sessions, and whether within-year changes differed between years (the interaction model).

We used GLMMs to examine the influence of local food abundance on natal dispersal timing and whether local food abundance differed between our 2 study years. We developed a set of candidate models and used AICc and maximum likelihood estimators in R to compare candidate models. The response variable was natal dispersal timing, and the explanatory variables were year, ectoparasite treatment (treated with diatomaceous earth or control), and the change in relative food abundance between the first and second trapping sessions at each nest.

We also used a GLMM to test whether ambient levels of ectoparasite abundance differed between our 2 study years. The response variable was the index of ectoparasite abundance, and the explanatory variables were year, region (random effect), and age at which the juvenile was captured (ectoparasite infestation may change with juvenile age). We used AICc and maximum likelihood estimators in R to assess candidate models. We only included nests that were not treated for ectoparasites in this comparison of ectoparasite loads between years.

We then used GLMMs to examine the influence of ectoparasites on natal dispersal timing. The response variable was natal dispersal timing and the explanatory variables were year, region (random factor), and ectoparasite abundance. We used AICc and maximum likelihood estimators to assess candidate models, and the model containing ectoparasite abundance was compared with two null models (S6 Table). We included all nests at which the radio-collared juvenile initiated dispersal in this analysis.

## Results

Natal dispersal timing varied widely among juvenile burrowing owls ($\bar{x}$ = 72.3 ± 2.4, range 44.0–123.5 days old, *n* = 63). Of the 63 juveniles, 23 were in the group that only received supplemental food, 12 received only diatomaceous earth, 15 received both food and diatomaceous earth, and 13 received neither treatment. We were able to document dispersal timing for radio-marked juveniles from 49% of nests that received food supplements compared to 29% of nests that did not receive food supplements (Fisher’s Exact Two-tailed Test, *P* = 0.017). We were able to document dispersal timing for radio-marked juvenile from 36% of nests that were treated with diatomaceous earth compared to 40% of nests that were not treated with diatomaceous earth (Fisher’s Exact Two-tailed Test, *P* = 0.630). As we expected, the timing of parental departure influenced natal dispersal timing (S1 Table, Step 1). The highest-ranking models in the first-stage of model selection all contained parental departure, and the model with only parental departure had the greatest weight. Hence, we included parental departure in all models in stage two of our analysis.

**Fig 1:**
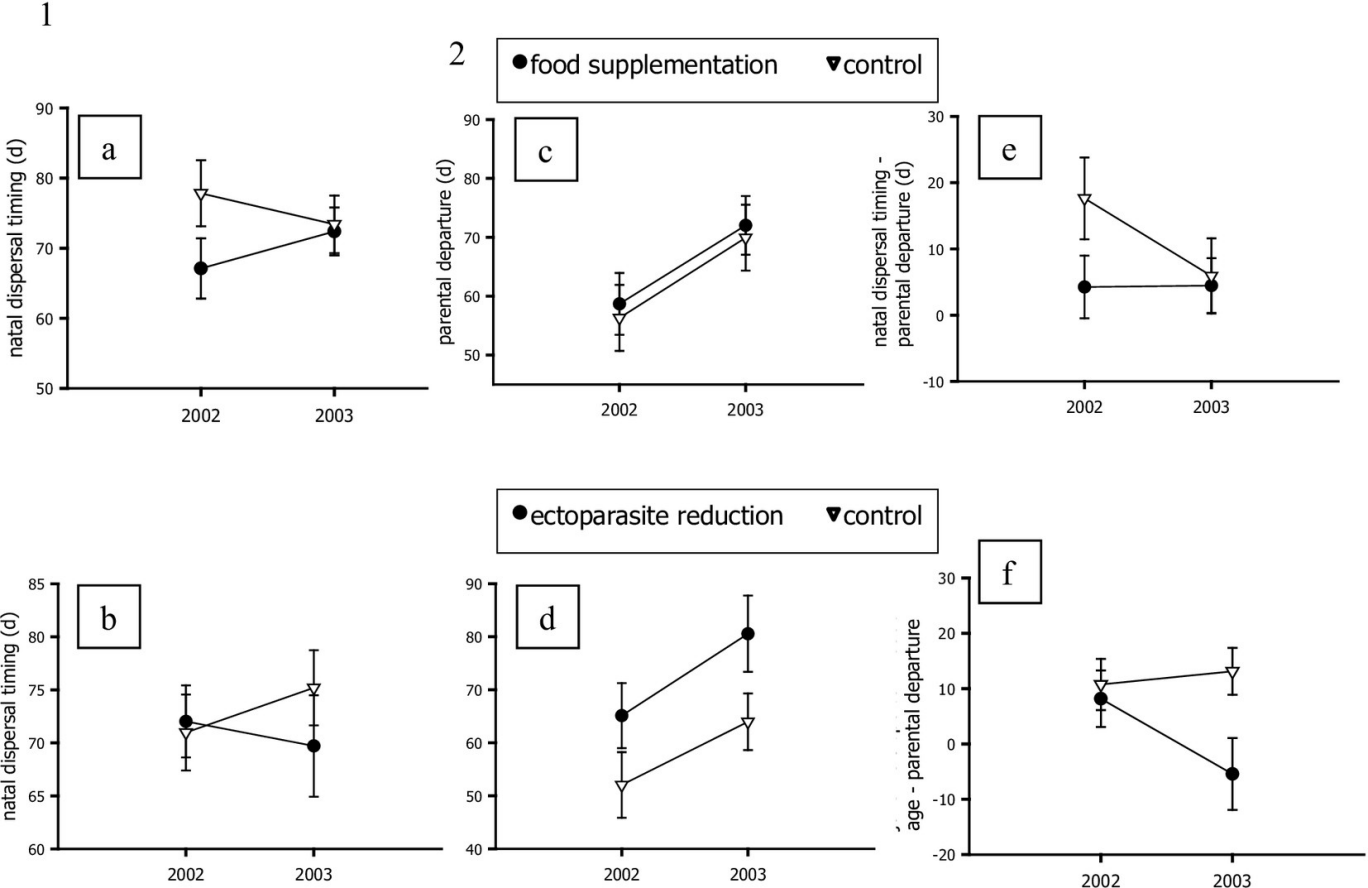
Model-averaged parameter estimates of the effects of food supplementation and ectoparasite reduction on natal dispersal timing in burrowing owls in Washington. Panel a) Effects of food supplementation on natal dispersal timing (age, in days, of the radio-marked juvenile when it departed the natal area). Candidate models are listed in S1 Table. Panel b) Effects of ectoparasite reduction on natal dispersal timing. Candidate models are listed in S1 Table. Panel c) Effects of food supplementation on parental departure (age, in days, of the radio-marked juvenile when its last parent departed the natal area). Candidate models are listed in S2 Table. Panel d) Effects of ectoparasite reduction on parental departure. Candidate models are listed in S2 Table. Panel e) Effects of food supplementation on the difference between natal dispersal timing and parental departure. Candidate models are listed in S3 Table. Panel f) Effects of ectoparasite reduction on the difference between natal dispersal timing and parental departure. Candidate models are listed in S3 Table.

Experimental manipulation of food and ectoparasites influenced natal dispersal timing in burrowing owls and the effects of the experimental treatments varied between the 2 years (S1 Table, Step 2). The model-averaged parameter estimates indicate that juveniles at nests that received food supplements initiated dispersal at a younger age than juveniles at control nests in 2002, but food supplements had no effect on natal dispersal timing in 2003 (interaction between food treatment*year in top model; Fig 1, Panel a). The effect of ectoparasite treatments also differed between the 2 study years (interaction between ectoparasite treatment*year in top model; S1 Table, Step 2). Ectoparasite reduction had no effect on natal dispersal timing in 2002, but the ectoparasite treatment did affect natal dispersal timing in 2003 (Fig 1, Panel b). Juveniles at nests where we experimentally reduced ectoparasites initiated dispersal at a younger age than juveniles at control nests.

Parental departure (the age of the radio-marked juvenile when the last of its 2 parents departed the natal area) was influenced by brood size (S2 Table, Step 1). In contrast to juvenile dispersal decisions, the experimental food supplementation did not affect when parents departed the natal area (Fig 1, Panel c). Although food supplementation treatment was in the third highest-ranked model and that model had <2.0 ΔAIC, the difference in deviance between the third- and first-ranked models was only 0.34 (S2 Table, Step 2), indicating that food supplementation did not contribute to improving model fit [66]. Parents departed the natal area when offspring were younger in 2002 compared to 2003 regardless of whether nests received food supplements or not (Fig 1, Panels c and d). However, parents at nests that were treated with diatomaceous earth delayed dispersal relative to parents at control nests in both years (S2 Table, Step 2; Fig 1, Panel d).

On average, juveniles dispersed from the natal area 8.0 ±2.6 days after their last parent departed the natal area. Brood size explained variation in the difference between natal dispersal timing and the age of juveniles when their last parent departed the natal area (S3 Table, Step 1). In other words, juveniles from larger broods were more likely to delay dispersal (relative to when their parents departed the natal area). Food and ectoparasite treatments affected when juveniles initiated dispersal relative to when their parents departed, but the effects differed between years (treatment*year interactions in all the top models; S3 Table, Step 2). Juveniles at nests that did not receive food supplements in 2002 initiated dispersal 17.6 ± 6.2 days after their last parent departed the natal area (Fig 1, Panel e), whereas food-supplemented juveniles in 2002 (and all juveniles in 2003) initiated dispersal much closer (4.2–5.9 ± 4.7–5.7 days) to the date that their last parent departed the natal area. And juveniles at nests that were treated with diatomaceous earth in 2003 initiated dispersal 5.4 ± 6.5 days before their last parent departed the natal area, whereas juveniles at nests not treated with diatomaceous earth in 2003 (and all juveniles in 2002) initiated dispersal 8.2–13.1 days after their last parent departed the natal area (Fig 1, Panel f).

Ninety-seven percent of all the small mammals that we caught were deer mice (*Peromyscus maniculatus*), which are a major component of burrowing owl diets in many parts of their breeding range [48, 71]. The second session of small mammal trapping (_date_ = 14 Aug ± 2 days) occurred when many juveniles were foraging independently [72] and initiating dispersal (mean dispersal date of control juveniles was 6 Aug ± 3 days). The seasonal trend in local food abundance differed between the two years; the model containing year, trapping session, and year*session was ranked highest. The top-ranked model had an evidence ratio of 133.1 compared with the second-highest ranked model. Based on predicted values, relative abundance of small mammals decreased between the two trapping sessions in 2002 (from 12.4 ± 3.0 to 5.0 ± 3.0 rodents captured/1.1025-ha grid), but increased in 2003 (from 9.5 ± 6.2 to 13.4 ± 6.2 rodents captured/1.1025-ha grid). We observed only weak evidence that variation in local food abundance explains variation in natal dispersal timing (S4 Table). The model-averaged estimate of the change in food index between the two trapping sessions indicates that increasing food from early to late summer is associated with earlier dispersal, but the 95% confidence interval included 0 (model-averaged estimate: -0.92, unconditional SE: 0.57, 95% unconditional confidence interval: -2.04–0.2).

We found little evidence that ambient ectoparasite loads differed between 2002 and 2003 after controlling for region and age of juvenile. Models with region alone, and those with both region and the age a juvenile was captured ranked higher than models that included only year, although all models received some support (S5 Table). We found some evidence that ectoparasite load was positively associated with natal dispersal timing after controlling for year and region (β_ectoparasite index_ = 6.0 ± 2.5; S6 Table), suggesting that juveniles with fewer ectoparasites initiated dispersal at a younger age.

## Discussion

Juvenile burrowing owls varied widely in the age they initiated dispersal (an almost 3-fold difference between the youngest and oldest dispersers). Food abundance and ectoparasites influenced juvenile’s dispersal decisions directly, independent of when their parents departed the natal area (and how food and ectoparasites influenced their parents’ departure). Juveniles typically departed their natal area soon after their last parent departed, but adjusted their departure based on food and ectoparasites. Moreover, food and ectoparasites influenced dispersal decisions of adult and juvenile owls differently.

Experimental manipulation of both food and ectoparasites altered natal dispersal timing of juvenile owls relative to their parents’ departure during each of the two years (food supplements influenced natal dispersal timing in 2002 and ectoparasite treatments influenced natal dispersal timing in 2003), indicating that juvenile burrowing owls exhibit phenotypic plasticity in this trait and those responses are not entirely dependent on parental dispersal decisions. We did not detect an interaction between supplemental food and ectoparasite treatments, suggesting that each experimental treatment influenced parental departure and juvenile dispersal decisions independently of the other (at least in the context of our experimental manipulations). However, we detected annual differences in the effects of our two experimental treatments on dispersal decisions because ambient food abundance differed substantially between the two years of our experimental study.

Annual differences in how animals respond to experimental food supplements are not surprising if local prey availability differs annually [also see 68, 73]. The extent of food limitation in predators often varies widely across years [74], and this variation in local food abundance undoubtedly affects the impact of experimental food supplements on dispersal decisions even in randomly-assigned experimental treatments. For example, food supplements had greater effects on productivity of Spanish imperial eagles (*Aquila adalberti*) and bearded vultures (*Gypaetus barbatus*) in low- versus high-quality territories [75].

Adult and juvenile owls responded differently to our experimental treatments. Our results suggest that juvenile burrowing owls disperse earlier (relative to their parents’ departure) when conditions are good (due to food supplements, higher prey abundance, experimental reductions in ectoparasites, or lower ectoparasite abundance in the landscape). For example, juveniles dispersed shortly after parents departed the natal area if they received food supplements in the low food year (2002) and regardless of food treatment in the high food year (2003). Also, juveniles at nests treated for ectoparasites dispersed at a younger age than juveniles at control nests in the high food year, and those with lower ectoparasite loads dispersed at a younger age than those with higher ectoparasite loads in both years. But parents responded differently. Parents remained at their breeding sites longer in the higher food year and at nests where ectoparasites had been experimentally reduced in both years (i.e., when local food abundance was higher and ectoparasites were experimentally reduced), but food supplements did not affect timing of parental departure. Juveniles with less food available may develop at a slower rate, and thus may have been forced to delay their dispersal. Indeed, body condition influenced probability of natal dispersal in greater flamingos (*Phoenicopterus roseus*), with individuals in good condition being more likely to disperse [76]. Dispersal decisions of adult burrowing owls may have been less affected by variation in food availability because they may be more efficient foragers than juveniles or because they have the option of curtailing parental care sooner when food abundance is low (i.e., forcing their offspring to depart).

Our results corroborate those from several other studies where birds initiated natal dispersal earlier when they were in better condition, more dominant, or had more access to critical resources [e.g., 20, 31, 77–79]. However, our results contradict those of several other studies which suggest that higher food is associated with delayed dispersal, and that when local food abundance is high, juveniles are under no pressure to disperse quickly and may benefit from the safe haven of their natal territory [17]. For example, experimentally decreased food caused early dispersal in western bluebirds (*Sialia mexicana*) [80]. Similarly, year-round supplemental food caused delayed dispersal in carrion crows (*Corvus corone corone*) [81]. However, many past studies did not control for the effects of experimental changes in food abundance on parental departure (i.e., could not distinguish between a direct or indirect effect of their treatments on juvenile dispersal). However, in Spanish imperial eagles, dispersal decisions were also somewhat independent of parents as evidenced by similarly long post-fledging dependence periods prior to dispersal between hacked birds (without parents present) and non-hacked birds [79]. The effect of food abundance on age of juvenile dispersal probably differs among species (and even among populations within species) based on migratory tendency, extent of philopatry of the adults, and species-specific breeding strategies. Additionally, the costs and benefits of dispersal may differ among species and those differences may explain the interspecific variation in response to food supplementation. For example, species in which juvenile mortality does not increase following dispersal away from the natal area, such as red-bellied woodpeckers (*Melanerpes carolinus)* [82], may initiate dispersal movements regardless of body condition. A comparative analysis across experimental studies is needed to help explain why changes in food abundance affect dispersal decisions in juvenile birds so differently across species.

The effect of the ectoparasite treatments also differed between years. Nestling burrowing owls are often infested with large numbers of ectoparasites [40, 42, 43], but little is known about the effect of these ectoparasites on burrowing owl behavior and demography, but see [83, 84] for the effects of plague in prairie dog (*Cynomys* spp.) colonies on burrowing owls. Cliff swallows (*Hirundo pyrrhonota*) and barn swallows (*Hirundo rustica*) more heavily infested with ectoparasites were more likely to disperse and departed the natal colony earlier in the nesting cycle than less-infested swallows and swallows at less-infested sites [85–88]. In contrast, great tit (*Parus major*) nestlings experimentally infested with ectoparasites had a prolonged nestling period [89] and initiated dispersal at an older age. Similarly, European pied flycatchers (*Ficedula hypoleuca*) with fewer ectoparasites were larger during the nestling period [90]. The causes and consequences of changes in natal dispersal timing clearly differ among species, and future research should seek to understand why some species disperse earlier when conditions are good while other species disperse later.

In our study, experimental reductions in ectoparasites affected the timing of dispersal movements in adult owls (treated adults departed when offspring were older compared to controls). In contrast, we found a positive correlation between ectoparasite loads and natal dispersal timing in juveniles, again suggesting that parents and offspring react differently to variation in ectoparasite loads. High ectoparasite loads may be detrimental to juvenile survival or recruitment if they cause parents to depart the natal area earlier than they normally would (as our results and those of others suggest, [85–88], forcing offspring to attain independence at a younger age.

Our results suggest that natal dispersal timing is a phenotypically plastic trait that is influenced by a suite of environmental factors. Plasticity in natal dispersal timing is likely beneficial because early dispersal is probably the preferred strategy under some environmental conditions and delayed dispersal the preferred strategy under others. The condition- and context-dependence suggested by our results may help explain why some past studies reported an effect of supplemental food on natal dispersal timing [4, 45, 91], whereas other studies found no effect [91, 92]. Indeed, past studies have reported both positive and negative relationships, with higher food or lower ectoparasites causing juveniles to initiate dispersal earlier in some studies [76, 77, 85, 93, 94], and later in others [19, 80, 93]. Moreover, parents’ behavior may influence natal dispersal timing, either via their own departure from the natal area (cessation of parental care), by forcing juveniles to depart (parent-offspring conflict) [21, 95], or by merely tolerating juvenile presence [96, 97]. What parents do may also differ depending on local conditions; parents may delay their own departure or be more tolerant of juveniles when conditions are better. Our results suggest that at least some of the differences across studies in the effect of supplemental food (and other experimental manipulations) on natal dispersal timing may be due to differences in resource availability in the landscape during the time of the experimental manipulations.

Understanding why species differ in their response to the same selective pressures will provide insights into the underlying causes of the diversity of life history strategies. For example, the best strategy may be to delay dispersal when environmental conditions are good if dispersal distance is short and many juveniles are typically recruited into the local breeding population. In contrast, the best strategy may be to initiate dispersal early when conditions are good if juveniles are typically not recruited into the local breeding population (as is typical of our study population). Indeed, behavioral dominance, superior physical condition, or better environmental conditions can result in either delayed or accelerated dispersal timing in birds depending on the species’ dispersal strategy [31, 98]. If resources (e.g., territory vacancies) are available on a first-come-first-serve basis to juveniles, dispersing as early as possible might be the best strategy and juveniles on the best territories can develop faster and initiate dispersal earlier than those on lower-quality territories [99]. If, on the other hand, scramble competition for resources does not occur among juveniles, then remaining on the natal territory longer and reaping the benefits of being in a familiar area may be the best strategy for juveniles on the best territories (but not perhaps for those on lower-quality territories) [100]. Novel next steps include studies designed to examine the effects of natal dispersal timing on post-dispersal survival and subsequent recruitment, and how those effects differ between migratory and non-migratory populations [e.g., 21, 31, 100, 101].

In an applied context, natural resource agencies often restrict disturbance around nest sites for species of management concern [102] and the duration of these restrictions are most effective if based, at least in part, on natal dispersal timing. These decisions are particularly important for rare or declining species of high conservation concern. Therefore, identifying the factors that influence natal dispersal timing may help improve regulatory policies to prevent actions during the post-fledging period that may cause juveniles to hasten dispersal. Understanding the suite of factors that influence natal dispersal timing may become even more important as our anthropogenic footprint expands. For example, climate change and changes in land-use may also influence the timing of natal dispersal and such changes may have potentially negative consequences. Thoroughly documenting these effects and the limits to species’ plasticity in response to landscape changes will be crucial if we hope to ensure species persistence in the face of future anthropogenic changes.

## Supporting information

S1 Table Comparison of models designed to explain intraspecific variation in natal dispersal timing (age [d] that dispersal was initiated) in juvenile burrowing owls in Washington. In Step 1, only covariates (not our main effects) were included in the candidate model set. R2 for the top model was 0.40. In Step 2, models include the covariate from the top model selected in the first stage (parental departure), our main effects (food and ectoparasite treatments), and year. R2 for the top model in Step 2 was 0.52. Only the models that had AICc weights ≥0.01 (and the null models) are presented.
(PDF)

S2 Table Comparison of models designed to explain intraspecific variation in parental departure (the age [d] of radio-marked juvenile burrowing owls when the last parent departed the natal area) in Washington. In Step 1, only covariates are included in the candidate model set. R2 for the top model was 0.08. In Step 2, models include the covariate from the top model selected in the first stage (brood), our main effects (food and ectoparasite treatments), and year. R2 for the top model in Step 2 was 0.23. Only the models that had AICc weights ≥0.01 (and the null models) are presented.
(PDF)

S3 Table Comparison of models designed to explain intraspecific variation in the difference between natal dispersal timing (age [d] that dispersal was initiated) and parental departure (age [d] of the juvenile when the last parent departed the natal area) in juvenile burrowing owls in Washington. In Step 1, only covariates are included in the candidate model set. *R*^2^ for the top model was 0.09. In Step 2, models include the covariate from the top model selected in the first stage (brood), our main effects (food and ectoparasite treatments), and year. *R*^2^ for the top model in Step 2 was 0.33. Only the models that had AICc weights ≥0.01 (and the null models) are presented.
(PDF)

S4 Table Comparison of models designed to explain intraspecific variation in natal dispersal timing (age [d] that dispersal was initiated) in juvenile burrowing owls for the subset of nests that did not receive supplemental food and at which we conducted small mammal trapping in Washington. (PDF)

S5 Table Comparison of models designed to explain intraspecific variation in ectoparasite levels of juvenile burrowing owls in Washington. (PDF)

S6 Table Comparison of models designed to explain intraspecific variation in natal dispersal timing (age [d] that dispersal was initiated) in juvenile burrowing owls in Washington. (PDF)

## References

[1] Penteriani V, Delgado MM. Thoughts on natal dispersal. Journal of Raptor Research 2009;43:90–98.

[2] Bonte D, Dahirel M. Dispersal: a central and independent trait in life history. Oikos 2017;126:472–479.

[3] Heinz SK, Wissel C, Frank K. The viability of metapopulations: individual dispersal behaviour matters. Landscape Ecology 2006;21:77–89.

[4] Kenward RE, Marcström V, Karlbom M. Post-nestling behaviour in goshawks, *Accipiter gentilis*: I. The causes of dispersal. Anim. Behav. 1993;46:365–370.

[5] Russell EM, Yom-Tov Y, Geffen E. Extended parental care and delayed dispersal: Northern, tropical, and southern passerines compared. Behav. Ecol. 2004;15:831–838.

[6] Ellsworth EA, Belthoff JR. Postfledging behavior of western screech-owls: the effect of dominance on timing of dispersal. J. Raptor Res. 1995;29:55–56.

[7] King RA, Belthoff JR. Post-fledging dispersal of Burrowing Owls in Southwestern Idaho: Characterization of movements and use of satellite burrows. Condor 2001;103:118–126.

[8] Emlen ST. The evolution of helping. I. An ecological constraints model. Am. Nat. 1982;119:29–39.

[9] Langen TA. Prolonged offspring dependence and cooperative breeding in birds. Behav. Ecol. 2000;11:367–377.

[10] Verhulst S. Multiple breeding in the Great Tit, II. The costs of rearing a second clutch. Funct. Ecol. 1998;12:132–140.

[11] Gruebler MU, Naef-Daenzer B. Postfledging parental effort in barn swallows: evidence for a trade-off in the allocation of time between broods. Anim. Behav. 2008;75:1877–1884.

[12] Mayer M, Zedrosser A, Rosell F. When to leave: the timing of natal dispersal in a large, monogamous rodent, the Eurasian beaver. Animal Behaviour 2017;123:375–382.

[13] Maag N, Cozzi G, Clutton‐Brock T, Ozgul A. Density‐dependent dispersal strategies in a cooperative breeder. Ecology 2018;99:1932–1941. doi: 10.1002/ecy.2433 29934962

[14] Ekman J, Dickinson JL, Hatchwell BJ, Griesser M. Delayed dispersal. In: Koenig WD, Dickinson JL (eds) Ecology and Evolution of Cooperative Breeding in Birds. Cambridge University Press, Cambridge; 2004. p. 35–47.

[15] Sparkman AM, Adams JR, Steury TD, Waits LP, Murray DL. Direct fitness benefits of delayed dispersal in the cooperatively breeding red wolf (*Canis rufus*). Behavioral Ecology 2010;22:199–205.

[16] Kokko H, Lundberg P. Dispersal, migration, and offspring retention in saturated habitats. Am. Nat. 2001;157:188–202. doi: 10.1086/318632 18707271

[17] Kokko H, Ekman J. Delayed dispersal as a route to breeding: territorial inheritance, safe havens, and ecological constraints. Am. Nat. 2002;160:468–484. doi: 10.1086/342074 18707523

[18] Gardner JL, Magrath RD, Kokko H. Stepping stones of life: Natal dispersal in the group-living but non-cooperative speckled warbler. Anim. Behav. 2003;66:521–530.

[19] Wiens DJ, Reynolds RT, Noon BR. Juvenile movement and natal dispersal of Northern Goshawks in Arizona. Condor 2006;108:253–269.

[20] Delgado MdM, Penteriani V, Revilla E, Nams VO. The effect of phenotypic traits and external cues on natal dispersal movements. J. Anim. Ecol. 2010;79:620–632. doi: 10.1111/j.1365-2656.2009.01655.x 20102419

[21] Tarwater CE, Brawn JD. Family living in a Neotropical bird: variation in timing of dispersal and higher survival for delayed dispersers. Anim. Behav. 2010;80:535–542.

[22] Vitz AC, Rodewald AD. Movements of fledgling Ovenbirds (*Seiurus aurocapilla*) and Worm-eating Warblers (*Helmitheros vermivorum*) within and beyond the natal home range. The Auk 2010;127:364–371.

[23] Gienapp P, Merilä J. Sex-specific fitness consequences of dispersal in Siberian jays. Behav. Ecol. Sociobiol. 2011;65:131–140.

[24] Ausprey IJ, Rodewald AD. Post-fledging dispersal timing and natal range size of two songbird species in an urbanizing landscape. The Condor 2013;115:102–114.

[25] Ronce O, Clobert J, Massot M. Natal dispersal and senescence. Proceedings of the National Academy of Sciences of the United States of America 1998;95:600–605. doi: 10.1073/pnas.95.2.600 9435238 PMC18466

[26] Bowler DE, Benton TG. Causes and consenquences of animal dispersal strategies: relating individual behaviour to spatial dynamics. Biol. Rev. 2005;80:205–225. 15921049 10.1017/s1464793104006645

[27] Nunes S, Holekamp KE. Mass and fat influence the timing of natal dispersal in Belding’s ground squirrels. Journal of Mammalogy 1996;77:807–817.

[28] Sarno RJ, Bank MS, Stern HS, Franklin WL. Forced dispersal of juvenile guanacos (Lama guanicoe): Causes, variation, and fates of individuals dispersing at different times. Behavioral Ecology and Sociobiology 2003;54:22–29.

[29] Matthysen E. Density-dependent dispersal in birds and mammals. Ecography, 2005;28:403–416.

[30] Cam E, Monnat JY, Hines JE. Long-term fitness consequences of early conditions in the kittiwake. J. Anim. Ecol. 2003;72:411–424.

[31] Middleton HA, Green DJ. Correlates of postfledging survival, the timing of dispersal, and local recruitment in American Dippers. Can. J. Zool. 2008;86:875–881.

[32] Todd LD. Dispersal patterns and post-fledging mortality of juvenile Burrowing Owls in Saskatchewan. J. Raptor Res. 2001;35:282–287.

[33] Haug EA, Millsap BA, Martell MS. Burrowing Owl (*Speotyto cunicularia*). In: Poole A, Gill F (eds) The Birds of North America, No. 61. The Academy of Natural Sciences and The American Ornithologists’ Union, Philadelphia, PA and Washington, D.C.; 1993.

[34] Conway CJ, Garcia V, Smith MD, Ellis LA, Whitney JL. Comparative demography of Burrowing Owls in agricultural and urban landscapes in southeastern Washington. J. Field Ornith. 2006;77:280–290.

[35] Conway CJ, Pardieck KL. Population trajectory of burrowing owls in eastern Washington. Northwest Science 2006;80:292–297.

[36] Macías-Duarte A, Conway CJ. Distributional changes in the Western Burrowing Owl (*Athene cunicularia hypugaea*) in North America from 1967 to 2008. Journal of Raptor Research 2015;49:75–83.

[37] Conway CJ. Spatial and temporal patterns in population trends and burrow usage of Burrowing Owls in North America. Journal of Raptor Research 2018;52:129–142.

[38] Macías-Duarte A, Conway CJ, Culver M. Agriculture creates subtle genetic structure among migratory and non-migratory populations of Burrowing Owls throughout North America. Ecology and Evolution 2020;10:10697–10708. doi: 10.1002/ece3.6725 33072290 PMC7548177

[39] Holroyd G, Trefry H, Duxbury J, Valdez E. Population dynamics, dispersal and conservation of the ‘Canadian’ burrowing owl, Athene cunicularia. Ardea 2009;97:645.

[40] Smith BW, Belthoff JR. Identification of ectoparasites on Burrowing Owls in Southwestern Idaho. J. Raptor Res. 2001;35:159–161.

[41] Skoruppa MK, Pearce B, Woodin MC, Hickman GC. Ectoparasites of burrowing owls (*Athene cunicularia hypugaea*) wintering in Southern Texas. Texas Journal of Science 2006;58:73–78.

[42] Graham CB, Eisen RJ, Belthoff JR. Detecting Burrowing Owl bloodmeals in *Pulex irritans* (Siphonaptera: Pulicidae). J. Med. Entomol. 2016;53:446–450. 26545716 10.1093/jme/tjv177 PMC5572895

[43] Belthoff JR, Bernhardt SA, Ball CL, Gregg M, Johnson DH, Ketterling R, et al. Burrowing Owls, *Pulex irritans*, and plague. Vector-Borne and Zoonotic Diseases 2015;15:556–564. 26367482 10.1089/vbz.2015.1772

[44] Freeman NE. Norris DR, Sutton AO, Newman AEM. Raising young with limited resources: supplementation improves body condition and advances fledging of Canada Jays. Ecology 2020; 101:e02909. doi: 10.1002/ecy.2909 31605623

[45] Gjerdrum C. Parental provisioning and nestling departure decisions: A supplementary feeding experiment in tufted puffins (*Fratercula cirrhata*) on Triangle Island, British Columbia. Auk 2004;121:463–472.

[46] Sprague RS, Breuner CW. Timing of fledging is influenced by glucocorticoid physiology in Laysan Albatross chicks. Hormones and Behav. 2010; 58:297–305. doi: 10.1016/j.yhbeh.2010.03.002 20223237

[47] Moreno J. 2022. Food supplementation of parents before hatching of the young prolongs the nestling period in European pied flycatchers (Ficedula hypoleuca). Ibis 2022; 164:1252–1256.

[48] Wellicome TI. Hatching asynchrony in Burrowing Owls is influenced by clutch size and hatching success but not by food. Oecologia 2005;142:326–334. doi: 10.1007/s00442-004-1727-8 15480800

[49] Conway M, Conway CJ, Nadeau CP. Intraspecific variation in reproductive traits of burrowing owls. Journal of Ethology 2012;30:395–402.

[50] Lundblad CG, Conway CJ. Nest microclimate explains intraspecific variation in avian life-history traits across latitudinal gradients. Ecology 2021;102:e03338. 33710621 10.1002/ecy.3338

[51] Smith MD, Conway CJ. Use of mammal manure by nesting burrowing owls: A test of four functional hypotheses. Anim. Behav. 2007;73:65–73.

[52] Blackwood JD. Eastside Draft Environmental Impact Statement. U.S. Forest Service and U.S. Bureau of Land Management, Walla Walla, WA, USA; 1997.

[53] Conway CJ, Simon JC. Comparison of detection probability associated with burrowing owl survey methods. J. Wildl. Manage. 2003;67:501–511.

[54] Marti CD. Food consumption and pellet formation rates in four owl species. Wilson Bull. 1973;83:178–181.

[55] Dawson RD. Efficacy of diatomaceous earth at reducing populations of nest-dwelling ectoparasites in Tree Swallows. J. Field Ornith. 2004;75:232–238.

[56] Belanger JD. The Complete Idiot’s Guide To Raising Chickens: Everything You Need to Know to Care for Your Own Flock of Chickens. Penguin Group, Inc., New York; 2010.

[57] Hsu CC, Chan MM, Wheler CL. Biology and Diseases of Chinchillas. Pages 387–409 in Laboratory Animal Medicine (Fox JG., Anderson LC, Otto GM, Pritchett-Corning KR, and Whary MT, eds.). Academic Press, Cambridge, MA; 2015.

[58] Thomsen L. Behavior and ecology of Burrowing Owls on the Oakland Municipal Airport. Condor 1971;73:177–192.

[59] Ronan NA. Habitat Selection, Reproductive Success, and Site Fidelity of Burrowing Owls in a Grassland Ecosystem. M.S. Thesis, Oregon State University, Corvallis, Oregon; 2002.

[60] Fisher RJ, Poulin RG, Todd LD, Brigham RM. Nest stage, wind speed, and air temperature affect the nest defence behaviours of burrowing owls. Can. J. Zool. 2004;82:724–730.

[61] Garcia V. Effects of food and ectoparasites on age of natal dispersal in burrowing owls M.S. Thesis, University of Arizona, Tucson, Arizona; 2005.

[62] Garcia V, Conway CJ, Ellis LA. Protocol for estimating burrowing owl reproductive parameters based on data recorded during repeated visits to occupied burrows. USGS Arizona Cooperative Fish and Wildlife Research Unit, Tucson, AZ; 2007.

[63] Conway CJ, Garcia V. Effects of radio transmitters on natal recruitment of burrowing owls. J. Wildl. Manage. 2005;69:404–408.

[64] Priest JE. Age identification of nestling burrowing owls. Journal of Raptor Research Report 1997;9:125–127.

[65] R Core Team. R: A language and environment for statistical computing. R Foundation for Statistical Computing, Vienna, Austria. http://www.R-project.org/; 2014.

[66] Anderson DR. Model-Based Inference in the Life Sciences: A Primer on Evidence. Springer, New York, NY, USA; 2008.

[67] Mazerolle MJ. AICcmodavg: Model selection and multimodel inference based on (Q)AIC(c) R package version 2.1–0; 2011.

[68] Poulin RG, Wellicome TI, Todd LD. Synchronous and delayed numerical responses of a predatory bird community to a vole outbreak on the Canadian prairies. J. Raptor Res. 2001;35:288–295.

[69] Wellicome TI. Effects of food on reproduction in Burrowing Owls (*Athene cunicularia*) during three stages of the breeding season. Ph.D. Dissertation, University of Alberta, Edmonton, Alberta; 2000.

[70] Green GA, Fitzner RE, Anthony RG, Rogers LE. Comparative diets of burrowing owls in Oregon and Washington. Northwest Sci. 1993;67:88–93.

[71] Longhurst WM. The summer food of Burrowing Owls in Costilla County, Colorado. Condor 1942;44:281–282.

[72] Davies JM, Restani M. Survival and movements of juvenile burrowing owls during the postfledging period. The Condor 2006;108:282–291.

[73] Wellicome TI, Todd LD, Poulin RG, Holroyd GL, Fisher RJ. Comparing food limitation among three stages of nesting: supplementation experiments with the burrowing owl. Ecology and Evolution 2013;3:2684–2695. doi: 10.1002/ece3.616 24567832 PMC3930041

[74] Harper GH, Marchant A, Boddington DG. The ecology of the hen flea *Ceratophyllus gallinae* and the moorhen flea *Dasypsyllus gallinulae* in nestboxes. J. Anim. Ecol. 1992;61:317–327.

[75] Ferrer M, Morandini V, Baguena G, Newton I. Reintroducing endangered raptors: A case study of supplementary feeding and removal of nestlings from wild populations. J. Appl. Ecol. 2017;55:1360–1367.

[76] Barbraud C, Johnson AR, Bertault G. Phenotypic correlates of post-fledging dispersal in a population of greater flamingos: the importance of body condition. J. Anim. Ecol. 2003;72:246–257.

[77] Alonso JC, Martin E, Alonso JA, Morales MB. Proximate and ultimate causes of natal dispersal in the great bustard *Otis tarda*. Behav. Ecol. 1998;9:243–252.

[78] Ellsworth EA, Belthoff JR. Effects of social status on the dispersal behaviour of juvenile western screech-owls. Anim. Behav. 1999;57:883–892. doi: 10.1006/anbe.1998.1050 10202096

[79] Muriel R, Ferrer M, Balbontín J, Cabrera L, Calabuig CP. Disentangling the effect of parental care, food supply, and offspring decisions on the duration of the postfledging period. Behav. Ecol. 2015;26:1587–1596.

[80] Dickinson JL, McGowan A. Winter resource wealth drives delayed dispersal and family-group living in Western bluebirds. Proc. R. Soc. Lond. B. Biol. Sci. 2005;272:2423–2428. doi: 10.1098/rspb.2005.3269 16243691 PMC1559973

[81] Baglione V, Canestrari D, Marcos JM, Ekman J. Experimentally increased food resources in the natal territory promote offspring philopatry and helping in cooperatively breeding carrion crows. Proc. R. Soc. Lond. B. Biol. Sci. 2006;273:1529–1535. doi: 10.1098/rspb.2006.3481 16777748 PMC1560324

[82] Cox AS, Kesler DC. Reevaluating the cost of natal dispersal: Post-fledging survival of Red-Bellied Woodpeckers. The Condor 2012;114:341–347.

[83] Alverson KM, Dinsmore SJ. Factors affecting Burrowing Owl occupancy of prairie dog colonies. The Condor 2014;116:242–250.

[84] Eads DA, Biggins DE. Plague bacterium as a transformer species in prairie dogs and the grasslands of western North America. Conserv. Biol. 2015;29:1086–1093. doi: 10.1111/cobi.12498 25817984

[85] Brown CR, Brown MB. Ectoparasitism as a cause of natal dispersal in cliff swallows. Ecology 1992;73:1718–1723.

[86] Brown CR, Brown MB. Ectoparasitism shortens the breeding season in a colonial bird. Royal Society Open Science 2015;2:140508. doi: 10.1098/rsos.140508 26064606 PMC4448812

[87] Loye JE, Carroll SP. Nest ectoparasite abundance and cliff swallow colony site selection, nestling development, and departure time. In: Loye JE, Zuk M (eds) Bird-Parasite Interactions: Ecology, Evolution, and Behaviour, vol 2. Oxford University Press, Oxford and New York; 1991. p. 222–241.

[88] Saino N, Romano M, Scandolara C, Rubolini D, Ambrosini R, Caprioli M, et al. Brownish, small and lousy barn swallows have greater natal dispersal propensity. Anim. Behav. 2014;87:137–146.

[89] Fitze PS. Life history and fitness consequences of ectoparasites. J. Anim. Ecol. 2004;73:216–226.

[90] Cantarero A, López-Arrabé J, Redondo AJ, Moreno J. Behavioural responses to ectoparasites in Pied Flycatchers *Ficedula hypoleuca*: an experimental study. J. Avian Biol. 2013;44:591–599.

[91] Bustamante J. Family break-up in Black and Red Kites *Milvus migrans* and *M*. *milvus*: Is time of independence an offspring decision? Ibis 1994;136:176–184.

[92] Redpath SM, Thirgood SJ, Leckie FM. Does supplementary feeding reduce predation of red grouse by hen harriers? J. Appl. Ecol. 2001;38:1157–1168.

[93] Arroyo BE, De Cornulier T, Bretagnolle V. Parental investment and parent-offspring conflicts during the postfledging period in Montagu’s harriers. Anim. Behav. 2002;63:235–244.

[94] Belthoff JR, Dufty AM Jr. Corticosterone, body condition and locomotor activity: A model for dispersal in screech-owls. Anim. Behav. 1998;55:405–415. doi: 10.1006/anbe.1997.0625 9480707

[95] Trivers RL. Parent-offspring conflict. Am. Zool. 1974;14:249–264.

[96] Eikenaar C, Richardson DS, Brouwer L, Komdeur J. Parent presence, delayed dispersal, and territory acquisition in the Seychelles warbler. Behav. Ecol. 2007;18:874–879.

[97] Ekman J, Griesser M. Why offspring delay dispersal: experimental evidence for a role of parental tolerance. Proc. R. Soc. Lond. B. Biol. Sci. 2002;269:1709–1713. doi: 10.1098/rspb.2002.2082 12204132 PMC1691083

[98] Nilsson JÅ, Smith HG. Early fledgling mortality and the timing of juvenile dispersal in the Marsh Tit *Parus palustris*. Ornis Scand. 1985;16:293–298.

[99] Lens L, Dhondt AA. Effects of habitat fragmentation on the timing of Crested Tit *Parus cristatus* natal dispersal. Ibis 1994;136:147–152.

[100] Ekman J, Bylin A, Tegelstrom H. Increased lifetime reproductive success for Siberian jay (*Perisoreus infaustus*) males with delayed dispersal. Proc. R. Soc. Lond. B. Biol. Sci. 1999;266:911–915.

[101] Green DJ, Cockburn A. Post-fledging care, philopatry and recruitment in brown thornbills. J. Anim. Ecol. 2001;70:505–514.

[102] Ganey JL, Dick JL. Habitat relationships of Mexican spotted owls: current knowledge. Pages 1–42 in United States Fish and Wildlife Service. Recovery plan for the Mexican spotted owl. Volume II. Albuquerque, New Mexico; 1995.

