# Supplementary material for: Experimental changes in food and ectoparasites affect dispersal timing in juvenile burrowing owls
Source: PLoS One. 2024 Jul 26;19(7):e0306660. doi: 10.1371/journal.pone.0306660 (PMC11280279; doi:10.1371/journal.pone.0306660)
Supplement: S2 Table — In Step 1, only covariates are included in the candidate model set. R2 for the top model was 0.08. In Step 2, models include the covariate from the top model selected in the first stage (brood), our main effects (food and ectoparasite treatments), and year. R2 for the top model in Step 2 was 0.23. Only the models that had AICc weights ≥0.01 (and the null models) are presented. (PDF) [file pone.0306660.s002.pdf]

Table S2.

Model[1]	K	ΔAICc	Weight	Cumulative weight	Log Likelihood
Step 1					
brood	3	0.00	0.53	0.53	-286.75
brood + hatch	4	1.93	0.20	0.73	-286.58
null model	2	2.66	0.14	0.86	-289.19
brood + hatch + brood*hatch	5	3.91	0.07	0.94	-286.38
hatch	3	4.29	0.06	1.00	-288.90
Step 2					
brood + year + ectoparasite	5	0.00	0.31	0.31	-281.05
brood + year + ectoparasite + ectoparasite*year	6	1.24	0.17	0.48	-280.45
brood + year + food + ectoparasite	6	1.76	0.13	0.61	-280.71
brood + year + food + ectoparasite + food*ectoparasite	7	2.20	0.10	0.72	-279.65
brood + year + food + ectoparasite + ectoparasite*year	7	3.00	0.07	0.79	-280.05
brood + ectoparasite	4	4.05	0.04	0.83	-284.26
brood + year + food + ectoparasite + food*year	7	4.15	0.04	0.87	-280.62
brood + year	4	4.56	0.03	0.90	-284.52
brood + year + food + ectoparasite + food*year + ectoparasite*year	8	5.55	0.02	0.92	-280.00
brood + food + ectoparasite	5	5.58	0.02	0.94	-283.84
brood + food + ectoparasite + food*ectoparasite	6	6.14	0.01	0.96	-282.89
brood + year + food + ectoparasite + food*year + ectoparasite*year + food*ectoparasite	9	6.33	0.01	0.97	-279.02
brood + year + food	5	6.61	0.01	0.98	-284.36
brood (informed null model)	3	6.75	0.01	0.99	-286.75

[1] Hatch = Julian hatch date of the radio-marked juvenile; brood = the number of broodmates 30 days after hatch. Food = food supplemented or control nest; ectoparasite = nest treated with diatomaceous earth to reduce ectoparasites or control nest; year = 2002 or 2003.
