# Supplementary material for: Experimental changes in food and ectoparasites affect dispersal timing in juvenile burrowing owls
Source: PLoS One. 2024 Jul 26;19(7):e0306660. doi: 10.1371/journal.pone.0306660 (PMC11280279; doi:10.1371/journal.pone.0306660)
Supplement: S4 Table — (PDF) [file pone.0306660.s004.pdf]

Table S4.

Model[1]	K	ΔAICc	Weight	Cumulative weight	Log Likelihood
region	3	0.00	0.43	0.43	-75.97
region + slope of food index	4	1.16	0.24	0.67	-74.87
region + year	4	2.80	0.11	0.78	-75.69
region + ectoparasite index + slope of food index	5	3.30	0.08	0.86	-73.98
region + ectoparasite index	4	3.36	0.08	0.94	-75.97
region + year + slope of food index	5	5.07	0.03	0.97	-74.86
region + year + ectoparasite index	5	6.66	0.01	0.99	-75.66
region + year + ectoparasite index + slope of food index	6	7.48	0.01	1.00	-73.75

[1]Region = subunit of study area (random variable); ectoparasite index = index of ectoparasite load on juvenile owls; slope of food index = difference between trapping sessions in number of small mammals captured at each nest; year = 2002 or 2003.
