# Supplementary material for: Experimental changes in food and ectoparasites affect dispersal timing in juvenile burrowing owls
Source: PLoS One. 2024 Jul 26;19(7):e0306660. doi: 10.1371/journal.pone.0306660 (PMC11280279; doi:10.1371/journal.pone.0306660)
Supplement: S5 Table — (PDF) [file pone.0306660.s005.pdf]

Table S5.

**Model**[1]	**K**	**ΔAICc**	**Weight**	**Cumulative weight**	**Log Likelihood**
region	3	0.00	0.33	0.33	-74.36
age caught + region	4	0.46	0.26	0.59	-73.42
year + region	4	1.23	0.18	0.76	-73.81
year + age caught + year*age caught + region	6	1.92	0.13	0.89	-71.67
year + age caught + region	5	2.15	0.11	1.00	-73.05

[1]Region = subunit of study area (random variable); age caught = the age of the juvenile when its ectoparasite level was measured; year = 2002 or 2003.
