# Supplementary material for: Experimental changes in food and ectoparasites affect dispersal timing in juvenile burrowing owls
Source: PLoS One. 2024 Jul 26;19(7):e0306660. doi: 10.1371/journal.pone.0306660 (PMC11280279; doi:10.1371/journal.pone.0306660)
Supplement: S6 Table — (PDF) [file pone.0306660.s006.pdf]

Table S6.

Model[1]	K	ΔAICc	Weight	Cumulative weight	Log Likelihood
year + region+ ectoparasite index	5	0.00	0.75	0.75	-270.72
year + region	4	3.10	0.16	0.91	-273.45
region	3	4.23	0.09	1.00	-275.16

[1]Region = subunit of study area (random variable); ectoparasite index = index of ectoparasite load on juvenile owls; year = 2002 or 2003.
